# Hidden blood loss and its influencing factors after minimally invasive percutaneous transpedicular screw fixation in thoracolumbar fracture

**DOI:** 10.1186/s12891-022-05938-x

**Published:** 2022-11-07

**Authors:** Xin Yue, Jing Zhang, Tianze Sun, Wentao Zhang, Ming Yang, Zhonghai Li

**Affiliations:** 1 Department of Orthopaedics, First Affiliated Hospital of Dalian Medical University, Dalian, People’s Republic of China; 2 Department of Orthopaedics, The Sixth People’s Hospital of Chengdu, Chengdu, People’s Republic of China; 3 Key Laboratory of Molecular Mechanism for Repair and Remodeling of Orthopaedic Diseases, Liaoning Province, Dalian, People’s Republic of China

**Keywords:** Hidden blood loss (HBL), Risk factors, Minimally invasive percutaneous transpedicular screw fixation (MIPTSF), Multiple regression analysis, Complication

## Abstract

**Background:**

Minimally invasive percutaneous transpedicular screw fixation (MIPTSF) is generally accepted as a minimally invasive treatment for thoracolumbar fracture. However, hidden blood loss (HBL) caused by this procedure is usually disregarded. This study aimed to investigate the amount of HBL and its influencing factors after MIPTSF in thoracolumbar fracture.

**Methods:**

Between October 2017 and December 2020, a total of 146 patients (106 males and 40 females, age range 21–59 years) were retrospectively examined, and their clinical and radiological data were recorded and analyzed. The Pearson or Spearman correlation analysis was used to investigate an association between patient’s characteristics and HBL. Multivariate linear regression analysis was performed to elucidate the related clinical or radiological factors of HBL.

**Results:**

A substantial amount of HBL (164.00 ± 112.02 ml, 40.65% of total blood loss (TBL)) occurred after transpedicular screw internal fixation. Multivariate linear regression analysis revealed that HBL was positively associated with TBL (*p* < .001), percentage of vertebral height loss (VHL) (*p* < .001), percentage of vertebral height restoration (VHR) (*p* < .001), numbers of fractured vertebrae (*P* = .013), and numbers of fixed vertebral segments (*P* = .002).

**Conclusion:**

A large amount of HBL was incurred in patients undergoing MIPTSF in thoracolumbar fracture. More importantly, TBL, percentage of VHL, percentage of VHR, the numbers of fractured vertebrae and fixed vertebral segments were independent risk factors for HBL.

## Introduction

The thoracolumbar spine is one of the area’s most commonly affected by spinal fractures. However, conventional open posterior pedicle screw fixation causes increased intraoperative bleeding, a higher infection rate, postoperative back pain, delayed functional rehabilitation, and aggravation of posterior ligamentous complex (PLC) injury [1–3]. With advances in surgical techniques and instrumentation, the percutaneous approach has been successfully applied for pedicle screw fixation to treat thoracolumbar fracture [4]. The percutaneous approach allows spine surgeons to insert pedicle screws and rods and to connect them percutaneously through small skin incisions. Moreover, this system avoids the disadvantages of conventional surgical treatment, minimizes soft tissue injury, reduces intraoperative blood loss, and results in better postoperative pain scores than other approaches [5, 6]. According to past clinical experience, MIPTSF is associated with a relatively low perioperative blood loss because of small incision, reduced muscular dissection, and short operative time [7]. But, the patients with thoracolumbar fractures tend to have a lower postoperative HB level than anticipated after surgery despite the apparently satisfactory perioperative management of blood loss. Previous studies examined only the volume of visible blood loss in the perioperative period. However, hidden blood loss (HBL) penetrating tissues, retained in a dead space, and lost due to hemolysis is often disregarded by orthopedic surgeons [8].

Hidden blood loss (HBL) is not usually recognized by general assessment because of its invisibility [9]. HBL may exacerbate postoperative hemoglobin drop, affect postoperative outcomes, such as medical complications, increased blood transfusion risks, and prolonged postoperative rehabilitation [10]. The issue of HBL has been noted in other fields of orthopedic surgery. The concept of HBL was first put forward by Sehat in 2000 [9]. Sehat et al. reported that the proportion of HBL was 50% of the TBL in total knee arthroplasty. Xu et al. reported the mean hidden loss calculated with our recommendable method was 362.8 ml and 47% of total loss in lumbar fusion surgery [11]. However, few studies have considered HBL in MIPTSF surgery during treatment of AO type A1-A3 thoracolumbar fractures with no neurological symptoms. Therefore, we retrospectively reviewed medical data of patients who underwent MIPTSF in our department in an attempt to evaluate HBL and identified the influencing factors of HBL.

## Materials and methods

### Patient population

**Fig. 1:**
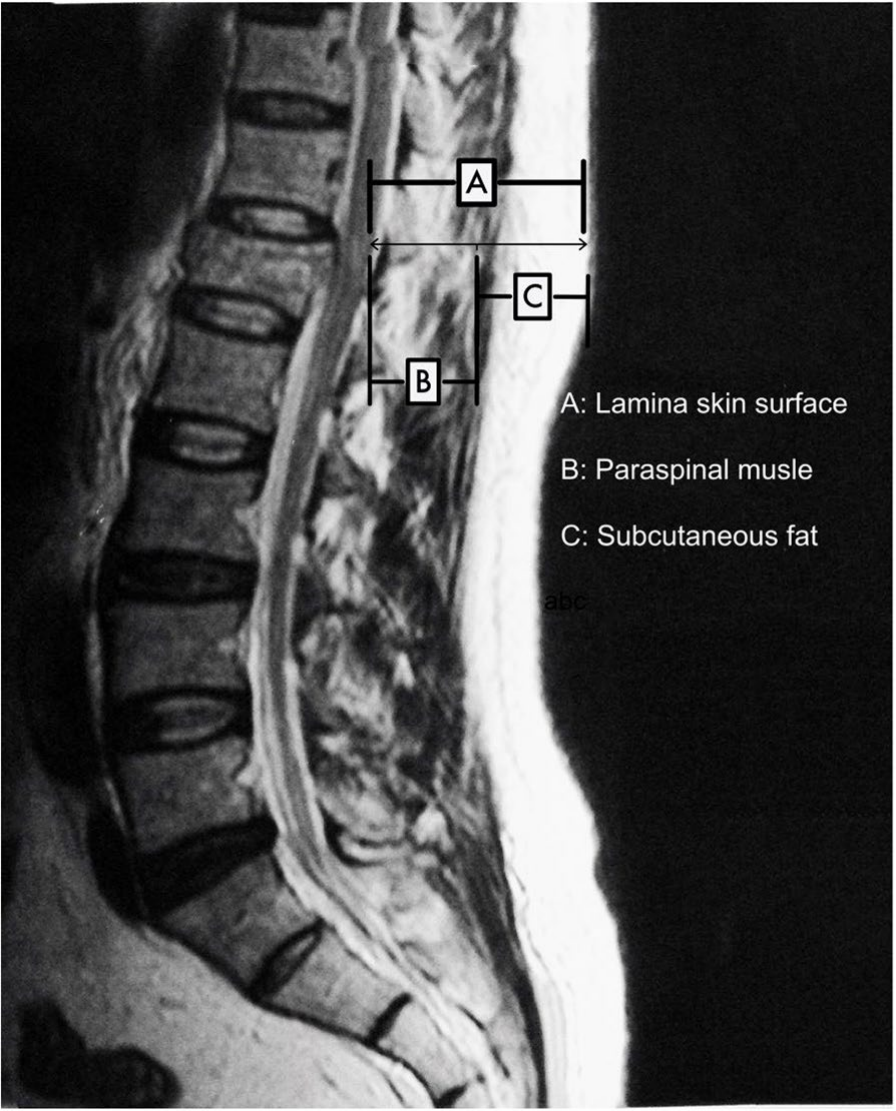
Diagram of the method used to measure thickness of the paraspinal muscles, subcutaneous fat, and lamina at the skin surface at the level of L1 using sagittal views was determined on T2-weighted MRI

This was a retrospective clinical study. The review of clinical database between October 2017 and December 2020 at one single center (First Affiliated Hospital of Dalian Medical University) was conducted. During the time period, more than 170 patients had received the same type of surgery in our institution. However, patients who strictly followed the inclusion and exclusion criteria and had complete perioperative data were included in the study. Finally, 146 patients aged 18 years or older who had AO type A1-A3 thoracolumbar fractures with radiographic evidence and hadn’t symptoms of nervous system damage treated by MIPTSF alone were included. Patient’s data were collected from the electronic medical records system of our institution. The information gathered including sex, age, height, weight, body mass index (BMI), hypertension (i.e., blood pressure ≥ 140/90 mmHg), diabetes mellitus (i.e., fasting blood-glucose ≥ 6.1 mmol/L), smoking, drinking, using hormones, combining with other fractures, low immunity, surgical duration, hospital stay, muscle thickness, subcutaneous fat thickness, muscle thickness/subcutaneous fat thickness, fracture classification, numbers of fracture segments, numbers of fixed vertebral segments preoperative and postoperative hematocrit (HCT and hemoglobin (HB), prothrombin time (PT), activated partial thromboplastin time (APTT), thrombin time (TT), fibrinogen, and platelet (PLT), percentage of vertebral height loss, percentage of vertebral height restoration. Preoperative magnetic resonance imaging (MRI) was used to determine the distance of the lamina from the skin surface, thickness of the paraspinal muscles, and thickness of the subcutaneous fat. These measurements were all performed at the level of L1 using sagittal views (Fig. 1). And pre-, intra-, postoperative findings were recorded as well. To prevent interobserver variability, imaging measurements were performed three times by two authors (MY and TZS), who was blinded to the operative details. To analyze intra- and inter-observer reliability values for imaging measurements, the same authors re-measured imaging parameters 4 weeks with the same protocol after the first measurements. We choose the average value as the final measurement result. All of the operations were performed by one spine surgeon with more than 20 years of working experience.

### Surgical technique

All patients were placed prone on the operation table with the free abdomen, and the standard general anesthesia was similar. Specifically, the mean arterial blood pressure monitored by a radial line was mainly 60 ~ 70 mmHg. The surgical technique was described as follows: The fracture segment was determined by the C-arm machine. After routine disinfection and placement of the drapes, the guide needles were placed in a satisfactory position, and fixation was performed using pedicle screws and connecting rods. The C-arm machine confirmed that the VHR recovered satisfactorily and the fixed position was good. The incisions were closed intermittently and no drainage device was installed.

### Inclusion and exclusion criteria

The inclusion criteria for the study were: (1) age of 18 ~ 60 years old, no sex preference, (2) A1-A3 thoracolumbar fractures (T11-L3); (3) surgical method: minimally invasive percutaneous transpedicular screw fixation. Our exclusion criteria were old thoracolumbar fractures (course > 3 months and radiography, CT, magnetic resonance imaging confirmed old fractures), spine infection, spinal cord compression syndrome, severe cardiopulmonary comorbidity, major coagulopathy (bleeding diseases caused by the deficiency or dysfunction of coagulation factors, including hemophilia, vitamin K deficiency, coagulation abnormalities caused by severe liver disease, antiphospholipid antibody syndrome and other diseases), and patients with symptoms of nervous system damage, liver cirrhosis or uremia.

### Management of blood loss

No patient received blood transfusion throughout the assessment period (from the day before the operation to the second or third day after the operation). All of the patients underwent a full blood count, including HCT, and HB before the surgery and 2 or 3 days after the surgery for calculation of blood loss. No drainage was typically placed in any of the patients. There was little visible blood loss after surgery, therefore, postoperative blood loss could be ignored.

### Calculation of hidden blood loss

Firstly, patient’s blood volume (PBV) was estimated in accordance with the formula of Nadler et al.[12]. PBV (L) = k1 × height(m)3 + k2 × weight(kg)2 + k3; where k1 = 0.3669, k2 = 0.03219 and k3 = 0.6041 for males, and k1 for females. = 0.3561, k2 = 0.03308 and k3 = 0.1833.

Secondly, according to the method of Gross et al. [13], the TBL was calculated based on the HCT level and the PBV, as follows: TBL (mL) = PBV (L) × (HCTpre – HCTpost) / HCTave, where HCTpre is the initial preoperative HCT, HCTpost is the HCT on the second or third day postoperatively, and HCTave is the average of the HCTpre and the HCTpost.

Finally, the method of Sehat et al. was used to calculate the HBL, as follows: HBL (mL) = TBL (mL) − VBL (mL) [9]*. Since n*o drainage was typically placed in any of the patients, intraoperative blood loss was equal to VBL, VBL was given by the measured suction loss and blood loss in swabs, and recorded by the anesthetists.

### The definition of anemia

According to the World Health Organization, anemia is characterized by HB levels of < 120 g/L for women and < 130 g/L for men) [14].

### Calculation of the percentage of vertebral height loss and restoration

**Fig. 2:**
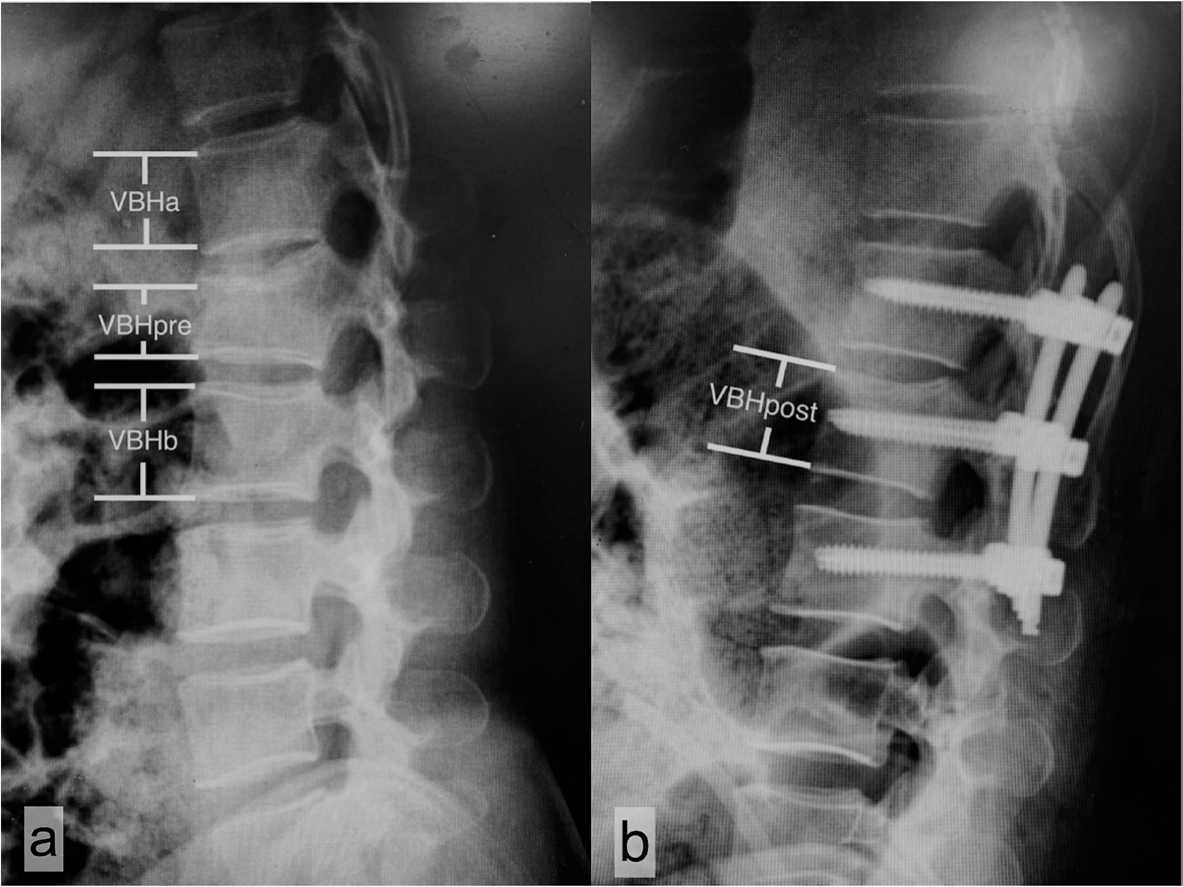
Diagram of the method for measuring the percentages of vertebral height loss (VHL) and vertebral height restoration (VHR) on sagittal plain radiograph. **a** Preoperative, (**b**) Postoperative

All of the included cases were examined using plain radiographs. The predicted height of each fractured vertebra was calculated according to the average height of the two adjacent vertebrae. And the anterior vertebral height loss and restoration was measured according to the affected vertebral body. The percentages of vertebral height loss (VHL) and vertebral height restoration (VHR) were calculated with the following equations [15]:

$$\mathrm{VBHave}=\left(\mathrm{VBHa}+\mathrm{ VBHb}\right) / 2$$

$$\mathrm{VHL }\left(\%\right)= \left(\mathrm{VBHave}-\mathrm{VBHpre}\right) / \mathrm{VBHave }\times 100\%$$

$$\mathrm{VHR }\left(\%\right)= \left(\mathrm{VBHpost}-\mathrm{VBHpre}\right) / \mathrm{VBHave }\times 100\mathrm{\%}$$

where VBHave is the average height of the 2 adjacent vertebrae, and VBHpre is the preoperative anterior vertebral body height, and VBHpost is the postoperative anterior vertebral body height. (Fig. 2).

### Statistical analysis

All of the independent variables were incorporated into the model using the method of “Enter.” Data analyses were performed with the SPSS 23.0 software. (International Business Machines Corporation, Armonk, NY). A chi-squared test was adopted to compare the preoperative and postoperative incidence of anemia. Pearson’s correlation (used for the continuous data), Spearman’s correlation analysis (used for the non-continuous data), and multivariate linear regression analysis were performed to evaluate the influencing factors associated with HBL. In all analyses, *P* < 0.05 was taken to indicate statistical significance.

## Results

**Fig. 3:**
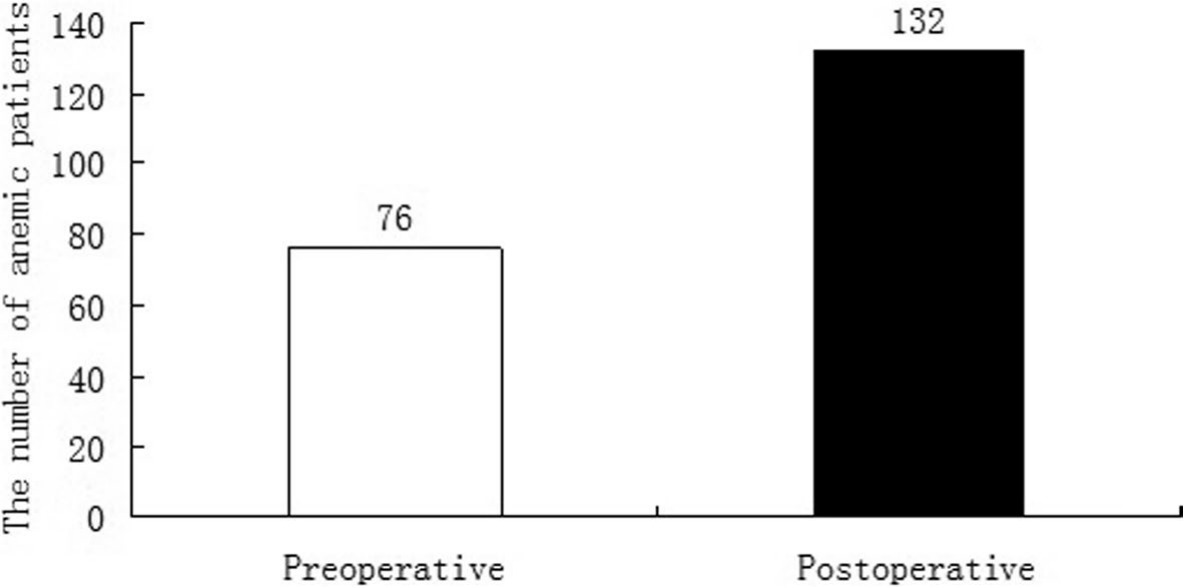
The number of anemic patients

A total of 146 patients were reviewed retrospectively in this study. Among these patients were 106 males and 40 females, with a mean age of 42.31 (range 21–59) years. Table 1 summarizes the demographic and clinical characteristics. The mean muscle thickness was 31.61 ± 7.84 mm, while the mean subcutaneous fat thickness was 19.84 ± 6.19 mm. The mean preoperative HCT and HB were 38.23 ± 4.41% and 124.86 ± 14.36 g/l. The mean postoperative HCT and HB were 35.18 ± 4.51% and 103.92 ± 13.67 g/l. The mean PBV was 4.87 ± 0.71L. The mean HBL was 164.00 ± 112.02 ml, 40.65% of TBL, indicating a considerable amount of HBL. The mean VBL was 239.45 ± 130.17 ml. The mean TBL was 403.45 ± 182.25 ml. 76 patients suffered from preoperative anemia, and 56 patients developed anemia after surgery (Fig. 3). There were significant differences between pre- and postoperative HCT (*P* < 0.001) and HB (*P* < 0.001) (Table 2).

**Table1:** Patients demographics

**Parameters**		**Statistics**
Total patients (n)		146
Sex (n)	Male	106
	Female	40
Age, yr		42.31 ± 7.90
BMI, kg/m2		25.33 ± 3.13
Muscle thickness, mm		31.61 ± 7.84
Subcutaneous fat thickness, mm		19.84 ± 6.19
Muscle thickness/Subcutaneous fat thickness		1.64 ± 0.26
Smoking (n)		35
Drinking (n)		17
Diabetes mellitus (n)		10
Hypertension (n)		13
Low immunity(n)		3
Using hormones(n)		8
Combining with other fractures(n)		20
Fracture classification(n)	A1	87
	A2	23
	A3	36
Preoperative HCT,%		38.23 ± 4.41
Postoperative HCT,%		35.18 ± 4.51
PBV, L		4.87 ± 0.71
TBL, ml		403.45 ± 182.25
VBL, ml		239.45 ± 130.17
VHL, %		45.58 ± 11.08
VHR, %		23.43 ± 9.47
Numbers of fractured vertebrae		1.18 ± 0.45
Numbers of fixed vertebral segments		3.03 ± 0.66
Hospital stay, d		12.28 ± 2.64
Surgical duration, min		120.14 ± 34.06
Preoperative Hb, g/l		124.86 ± 14.36
Postoperative Hb, g/l		103.92 ± 13.67
PT, s		11.46 ± 1.22
APTT, s		32.90 ± 6.18
TT, s		17.65 ± 1.25
Fibrinogen, g/l		3.37 ± 0.80
PLT, 109 /l		260.33 ± 66.39

*BMI* Body mass index, *HCT* Hematocrit, *PBV* Patient’s blood volume, *TBL* Total blood loss, *VBL* Visible blood loss, *VHL* Vertebral height loss, *VHR* Vertebral height restoration, *HB* Hemoglobin, *PT* Prothrombin time, *APTT* Activated partial thromboplastin time, *TT* Thrombin time, *PLT* Platelet

**Table 2:** Changes in HCT and HB level following MIPTSF

Parameters	Mean	SD	T	P
Preoperative and postoperative HCT, %	3.0486	1.3208	27.890	< .001
Preoperative and postoperative HB, g/L	20.945	11.672	21.683	< .001

*MIPTSF* Minimally invasive percutaneous transpedicular screw fixation, *HCT* Hematocrit, *HB* Hemoglobin, *SD* Standard deviation

The Pearson or Spearman correlation analysis for HBL found the following parameters with a *P* < 0.05 (Table 3): TBL (*P* = 0.000), BMI (*P* = 0.000), muscle thickness (*P* = 0.000), subcutaneous fat thickness (*P* = 0.000), surgical duration (*P* = 0.000), PT (*P* = 0.000), APTT (P = 0.000), TT (*P* = 0.038), diabetes mellitus (*P* = 0.048), fracture classification (type A1-A3) (*p* < 0.001), percentage of vertebral height loss (*p* < 0.001), percentage of vertebral height restoration (*p* < 0.001), numbers of fractured vertebrae (*p* < 0.001), and numbers of fixed vertebral segments (*p* < 0.001).

**Table 3:** Results of the Pearson or Spearman correlation analysis for HBL

Parameters	Sig	P
Sex	-.102	.219
Age	-.051	.539
BMI	.455	< .001
Muscle thickness	.778	< .001
Subcutaneous fat thickness	.646	< .001
Muscle thickness/Subcutaneous fat thickness	-.106	.204
Smoking	.036	.670
Drinking	.001	.993
Diabetes mellitus	-.164	.048
Hypertension	-.077	.357
Low immunity	-.037	.655
Using hormones	-.043	.608
Combining with other fractures	-.145	.081
Fracture classification	.519	< .001
PBV	.016	.852
TBL	.706	< .001
VBL	.128	.125
VHL	.938	< .001
VHR	.921	< .001
Numbers of fractured vertebrae	.625	< .001
Numbers of fixed vertebral segments	.746	< .001
Hospital stay	-.061	.466
Surgical duration	.356	< .001
PT	-.323	< .001
APTT	.590	< .001
TT	.172	.038
Fibrinogen	-.040	.629
PLT	-.074	.375

*HBL* Hidden blood loss, *BMI* Body mass index, *PBV* Patient’s blood volume;, *TBL* Total blood loss, *VBL* Visible blood loss, *VHL* Vertebral height loss, *VHR* Vertebral height restoration, *PT* Prothrombin time, *APTT* Activated partial thromboplastin time, *TT* Thrombin time, *PLT* Platelet

Next, we performed multiple and stepwise linear regression analysis to explore the association between HBL and the influential factors mentioned earlier. The TBL (*p* < 0.001), percentage of vertebral height loss (*p* < 0.001), percentage of vertebral height restoration (*p* < 0.001), numbers of fractured vertebrae (*P* = 0.013), and numbers of fixed vertebral segments (*P* = 0.002) were independent risk factors for HBL (Table 4). The results indicated that other factors were not significantly correlated with HBL.

**Table 4:** Results of multivariate linear regression for HBL

Parameters	Unstandardized		Standardized	t	P
	**β**	**SE**	**Β**		
Constant	-238.468	48.114		-4.956	< .001
TBL	.085	.018	.139	4.735	< .001
VHL	4.353	.582	.431	7.479	< .001
VHR	3.823	.669	.323	5.713	< .001
Numbers of fractured vertebrae	20.057	7.954	.081	2.522	.013
Numbers of fixed vertebral segments	19.468	6.184	.114	3.148	.002
BMI	.352	.925	.010	.380	.704
Muscle thickness	.859	.866	.060	.992	.323
Subcutaneous fat thickness	-1.184	.893	-.065	-1.326	.187
Surgical duration	-.134	.085	-.041	-1.578	.117
PT	-.632	2.184	-.007	-.289	.773
APTT	-.704	.534	-.039	-1.318	.190
TT	1.665	1.980	.019	.841	.402
Fracture classification (A2)	-5.512	7.073	-.018	-.779	.437
Fracture classification (A3)	10.699	8.057	.041	1.328	.187

*HBL* Hidden blood loss, *TBL* Total blood loss, *VHL* Vertebral height loss, *VHR* Vertebral height restoration, *BMI* Body mass index, *PT* Prothrombin time, *APTT* Activated partial thromboplastin time, *TT* Thrombin time

## Discussion

Studies on HBL after orthopedic surgery have mostly focused on total hip arthroplasty (THA), total knee arthroplasty (TKA), and ALIF/PLIF surgery [16]. In a work on anterior/posterior lumbar fusion surgery (ALIF/PLIF), HBL was approximately 40% of TBL [10, 16]. Chen et al. [17] reviewed and analyzed of the patients undergoing conventional posterior open approach, the average HBL was 382 ± 153.8 mL; and the average HBL of patients undergoing percutaneous approach was 240.0 ± 65.1 mL. In our study, a substantial amount of HBL (164.00 ± 112.02 ml, 40.65% of TBL) occurred after MIPTSF., the obtained amount was much greater than that of visible intraoperative blood loss. Some studies suggest that for patients undergoing total hip replacement, HBL is positively correlated with changes in BMI, blood transfusion, incision length, preoperative and postoperative HCT, and negatively correlated with age [18]. Nevertheless, there have been no previous studies regarding the influential factors correlated to the HBL during the MIPTSF of AO type A1-A3 thoracolumbar fractures. In this study, we investigated and identified the risk factors of HBL following this surgery by multivariate linear regression analysis. The results proposed that the TBL, percentage of vertebral height loss, percentage of vertebral height restoration, numbers of fractured vertebrae, and numbers of fixed vertebral segments were positive independent risk factors for HBL.

Our statistical analysis showed that the patients who had massive TBL suffered from more HBL than those who have little TBL. TBL was the independent risk factor, which may have to do with PBV, because TBL is calculated by multiplying PBV by changes of HCT and subtracting the IBL according to the Gross formula [13],which might relate to the patient’s weight and height. However, BMI had not been identified as a risk factor in our study, although body mass index was also calculated by weight and height. Based on collected data in our study, it was easy to find that HBL is directly related to a large amount of blood loss.

HBL during orthopedic surgery is generally accepted as being due to blood infiltration into tissue compartments and loss due to hemolysis [19, 20]. Our study found that the percentage of vertebral height loss and the percentage of vertebral height restoration were correlated with HBL. Vertebra involves cancellous bone, and its blood supply is abundant. The expansion of vertebral cavity will cause internal bleeding. The recovery of fractured vertebral body height may lead to enlarged cavity, and the space around vertebral body may be enlarged. We suspect that the blood would seep into these fracture spaces, leading to an increase in HBL [21]. Vertebral cavity and muscle space also provide storage cavity for HBL.

In our study, the numbers of fractured vertebrae and numbers of fixed vertebral segments were positively related to HBL, as Chen et al. guessed [17]. A previous study proposed that he number of fractured vertebrae was the risk factor of HBL in percutaneous kyphoplasty surgery [22]. Ju et al. held that ALIF was associated with substantial perioperative HBL, and the inclusion of L4/5 in the procedure were significant risk factors for increased blood loss [16]. However, we found that the number of fixed segments was an independent risk factor for hidden blood loss, and the fracture level was not included in our data. We will further explore the relationship between fracture level and hidden blood loss in the future.

Our previous studies had shown that muscle thickness is also an independent risk factor for hidden blood loss in spinal surgery [23], thicker muscle may be associated with larger penetrable tissue compartments, allowing blood to ooze into the tissue cavity [24]. Jiang et al. [25] found that posterior cervical soft tissue was positively correlated with both TBL and HBL in the expansive open-door laminoplasty (EOLP). But the muscle thickness or subcutaneous fat thickness was not clarified as a risk factor in this study. We think that this might be related to the less muscle damage caused by minimally invasive surgery. Therefore, we still need to further study the relationship between muscle thickness and HBL in the setting of spine surgery.

Excessive blood loss can increase the possibility of blood transfusion, which is associated with transfusion reactions, anaphylactic reaction, infections and delayed recovery [26]. In our study, no patient received blood transfusion throughout the assessment period, but there were still a portion of the patients received blood transfusions during hospitalization for anemia. Furthermore, excessive blood loss can prolong the hospitalization time and increase the use of medication [27]. The TBL, percentage of vertebral height loss, percentage of vertebral height restoration, numbers of fractured vertebrae, and numbers of fixed vertebral segments should be correctly understood before operation to ensure the safety of patient treatment.

## Study limitations

This study had some limitations. First of all, this was a retrospective study of a single center with few factors included in the analysis. Some factors that had an impact on HBL may be overlooked. Secondly, when we calculated blood loss, the blood volume was assumed to be constant throughout the perioperative period, but this might not be completely consistent with the actual situation. Thirdly, the HBL was calculated based on HCT on the second or third day after the operation. However, if fluid shifts were not complete at this time, the HBL obtained would be falsely low. After the assessment period, there were still a portion of the patients received blood transfusions during hospitalization for anemia. In addition, the number of patients included in this study was relatively small. As such, the results will be more reliable in future prospective studies with a large number of patients.

## Conclusions

Consequently, MIPTSF is associated with substantial HBL. More importantly, the total blood loss, percentage of vertebral height loss, percentage of vertebral height restoration, numbers of fractured vertebrae, and numbers of fixed vertebral segments were independent risk factors for HBL. It is important for surgeons to be aware of HBL, to avoid complications related to blood loss. Accurate perioperative HBL assessment can help prevent complications and improve rehabilitation.

## Data Availability

The data that support the findings of this study are available from ZHL, but restrictions apply to the availability of these data, which were used under license for the current study, and so are not publicly available. Data are however available from the authors upon reasonable request and with permission of ZHL.

## Abbreviations

MIPTSFM: Minimally invasive percutaneous transpedicular screw fixation
PLC: Posterior ligamentous complex
PBV: Patient’s blood volume
VBL: Visible blood loss
HBL: Hidden blood loss
TBL: Total blood loss
HCT: Hematocrit
HB: Hemoglobin
PT: Prothrombin time
APTT: Activated partial thromboplastin time
TT: Thrombin time
PLT: Platelet
MRI: Magnetic resonance imaging
VHL: Vertebral height loss
VHR: Vertebral height restoration
THA: Total hip arthroplasty
TKA: Total knee arthroplasty
ALIF: Anterior lumbar fusion
PLIF: Posterior lumbar fusion
